# Sun protection education for adolescents: a feasibility study of a wait-list controlled trial of an intervention involving a presentation, action planning, and SMS messages and using objective measurement of sun exposure

**DOI:** 10.1186/s12889-020-8265-0

**Published:** 2020-01-30

**Authors:** Gill Hubbard, John Cherrie, Jonathan Gray, Richard G. Kyle, Amanda Nioi, Charlotte Wendelboe-Nelson, Hilary Cowie, Stephan Dombrowski

**Affiliations:** 1 Department of Nursing and Midwifery, University of the Highlands and Islands, Centre for Health Sciences, Old Perth Road, Inverness, IV2 3JH UK; 2 Heriot-Watt University, Institute of Biological Chemistry, Biophysics and Bioengineering, Edinburgh, EH14 3AS UK; 3 Research Division, Institute of Occupational Medicine, Edinburgh, EH14 4AP UK; 4 School of Health & Social Care, Edinburgh Napier University, Sighthill Court, Edinburgh, EH11 4BN UK; 5 Faculty of Kinesiology, University of New Brunswick, Fredericton, Canada

**Keywords:** Skin cancer, Skin self-examination, Adolescence

## Abstract

**Background:**

People increase their risk of melanoma unless they are protected from the harmful effects of sun exposure during childhood and adolescence. We aimed to assess the feasibility of a three-component sun protection intervention- presentation, action planning, and SMS messages - and trial parameters.

**Methods:**

This feasibility wait-list trial was conducted in the United Kingdom in 2018. Students aged 13–15 years were eligible. Feasibility outcomes were collected for recruitment rates; data availability rates for objective measurements of melanin and erythema using a Mexameter and self-reported sunburn occurrences, severity and body location, tanning, sun protection behaviours and Skin Self-Examination (SSE) collected before (baseline) and after the school summer holidays (follow-up); intervention reach, adherence, perceived impact and acceptability. Quantitative data were analysed using descriptive statistics; qualitative data were analysed thematically.

**Results:**

Five out of eight schools expressing an interest in participating with four allocated to act as intervention and one control. Four parents/carers opted their child out of the study. Four hundred and eighty-seven out of 724 students on the school register consented to the study at baseline (67%). Three hundred and eighty-five were in intervention group schools. Objective skin measurements were available for 255 (66%) of the intervention group at baseline and 237 (61%) of the group at follow up. Melanin increased; erythema decreased. Complete self-report data were available for 247 (64%) students in the intervention group. The number of students on the school register who attended the presentation and given the booklet was 379 (98%) and gave their mobile phone number was 155 (40%). No intervention component was perceived as more impactful on sun protection behaviours. Adolescents did not see the relevance of sun protection in the UK or for their age group.

**Conclusions:**

This is the first study to use a Mexameter to measure skin colour in adolescents. Erythema (visible redness) lasts no more than three days and its measurement before and after a six week summer holiday may not yield relevant or meaningful data. A major challenge is that adolescents do not see the relevance of sun protection and SSE.

**Trial registration:**

International Standard Randomised Controlled Trial Number ISRCTN11141528.

Date registered 0/2/03/2018; last edited 31/05/2018. Retrospectively registered.

## Background

### Melanoma risk in adolescence

Sun exposure is important for health; it is involved in vitamin D synthesis and may induce feelings of well-being [1]. Sun *over*exposure is detrimental to health; 86% of melanoma cases in the UK are caused by overexposure to ultraviolet radiation (UVR) [2]. Overexposure leads to both DNA damage and immunosuppression, which mediate carcinogenesis [3]. In the last decade, melanoma incidence rates have increased by 50% in the UK and are projected to rise by 7% between 2014 and 2035, to 32 cases per 100,000 people by 2035 (https://www.cancerresearchuk.org/health-professional/cancer-statistics/statistics-by-cancer-type/melanoma-skin-cancer#heading-Zero). The estimated cost to the National Health Service (NHS) due to skin cancer will amount to over £180 million per annum in 2020 [4]. Evidence from meta-analyses show that melanoma risk is more closely linked with intermittent exposure to high-intensity sunlight than to chronic sunlight exposure [5]. Another meta-analysis found that an increased risk of melanoma was seen with increasing number of sunburns for all time-periods (childhood, adolescence, adulthood, and lifetime) [6]. Melanoma risk is increased regardless of whether sunburn occurs in childhood or adulthood [5–7]. However, adolescence is a key period for increasing melanoma risk; there is a greater propensity for sunburn during adolescence than childhood [8–11] or adulthood [12]. Several reports indicate that there is a steady decline in sun protection behaviours from childhood to adolescence [13, 14]. UK studies show that 51% of adolescents experience sunburn in the summer [15] and 44% of adolescents do not use sunscreen [16]. Health in adulthood has antecedents in childhood [17–19]; for example, behaviours (e.g. sunbathing) and attitudes (e.g. pro-tanning) associated with skin cancer emerge in adolescence and track into adulthood [20, 21]. Adolescence therefore provides a critical window of opportunity for the primary prevention of skin cancer caused by sunburn across the life-course.

### Sun protection interventions targeting adolescents

Theories inform what cognitions and emotions need to be addressed in behaviour change interventions. The Common-Sense Model of illness representation and self-regulation (CSM) [22] and Health Action Process Approach (HAPA) [23] informed the intervention being tested in this feasibility study. The CSM suggests that an ‘illness representation’ (e.g., skin cancer), has four dimensions: the *cause* dimension represents beliefs regarding the factors that are responsible for causing the illness (e.g., sunburn causes skin cancer), the *consequence* dimension refers to beliefs regarding the impact of an illness on overall quality of life (e.g., I will die if I get skin cancer), illness *identity* refers to beliefs about the illness label and knowledge about its symptoms (e.g., a mole that changes shape is a sign of skin cancer), the *timeline* dimension refers to beliefs about the time-scale or course of the illness (e.g., if I detect skin cancer early I will receive treatment and be cured) [24]. Expanded versions of the CSM include additional dimensions such as *risk perception* (e.g., people my age are not at risk of skin cancer) [25] and *controllability* (e.g., I can reduce my risk of skin cancer by using sunscreen) [26]. CSM is different to other health and risk behaviour models by explicitly recognising that people make simultaneous cognitive and emotional representations of an illness [24]. Thus, interventions using CSM ought to incorporate content that evoke an emotional response. According to HAPA, behavioural intentions are more likely to be translated into action when people generate specific plans [27]. Hence, sun safe interventions using HAPA will include planning to protect oneself from sun overexposure. Our previous study suggests that CSM and HAPA constructs are associated with sun protection behaviours [28] and other studies have also shown that illness representations [28, 29], risk perceptions [28, 30–33], and action planning [28, 30] are associated with sun protection behaviours in adolescence. A systematic review examining the efficacy of appearance-based interventions concluded that they generally produce positive effects of sun protection behaviours [34], which suggests that appearance beliefs is another important dimension to address in sun protection interventions. However, it is not known which psychosocial constructs and risk behaviour models have most explanatory power for sun protection behaviours in adolescence.

Education interventions involve imparting knowledge and developing sun protection skills [35]. Some forms of delivery of an education intervention are likely to be more acceptable to the target audience than others and may influence intervention adherence and thereby effectiveness [36]. Smartphone technology is a form of delivery that offers opportunities to deliver sun protection information using text messaging (also called short messaging service (SMS)) because of high levels (83%) of ownership in the target age group [37]. Moreover, this form of delivering sun protection information may represent a practical and cost-effective approach relative to interventions that are delivered in-person. A systematic review of eight studies concluded that the use of SMS and similar electronic technology improves sun protection behaviours [38]. However, only one included study involved adolescents, which was a pilot study with no control group of 113 adolescents (11–14 years) who received 36 text messages [39]. The study reported significant increases in self-reported wearing of sunscreen, hats and sunglasses [39].

A general criticism of evidence reporting the effect of sun protection interventions is reliance on self-report. People may present a favourable image of themselves on questionnaires, which is called socially desirable responding [40]. As several systematic reviews have highlighted, a major limitation of previous sun protection intervention studies is lack of objective measurement of sun protection behaviour and clinically-related proximal targets such as, sunburn occurrence [38, 41].

### Aims

The feasibility study of an educational sun protection intervention reported in this manuscript was designed to address limitations of our previous education intervention study [28]. The previous intervention was delivered in-person and sun protection behaviours were self-reported. Hence, the main purpose of this study was to assess the feasibility and acceptability of a sun protection education intervention with an additional intervention component – Short Messaging System (SMS). A further aim was to evaluate trial parameters such as, recruitment, use of a wait-list controlled trial design, and objective measurement of skin colour (erythema and melanin). The feasibility study was not powered to measure effect; rather, the purpose was to observe changes in potential outcomes of interest, and in particular, if outcomes changed in the intended direction. We do not report effectiveness data because it is generally recommended that feasibility and pilot studies descriptively evaluate a trial’s feasibility, acceptability and safety rather than test the effectiveness hypotheses of the planned main large-scale trial [2, 16–18, 42–45]. This is because the small amount of effect data available in feasibility and pilot studies means the degree of uncertainty is such that the chance of reaching inaccurate conclusions about intervention effect is high. Hence, robust and rigorous assessment of an intervention’s therapeutic implications must await adequately sized definitive pivotal trials [42]. Hence, recruitment and data completion rates are reported for intervention and control schools but outcomes (e.g., objective measure of skin colour) are only reported for the intervention school.

## Methods

### Design

We conducted a feasibility study of a wait-list controlled trial, with five schools allocated by the research team to an intervention group and one school to a wait-list control group. The last school to be recruited was allocated to the control group. We did not use randomisation to reduce bias in this feasibility study because we were not aiming to measure effect; rather, the purpose was to estimate important parameters that are needed to design the main trial so that the future main trial is internally and externally valid. The study was conducted in the United Kingdom between June and September in 2018 to deliberately coincide with summertime when risk of overexposure to the sun is greatest. University of the Highlands and Islands Research and Ethical Committee approved the study (REF: OLETHSHE1004).

### Study population and recruitment

The criteria for inclusion in the study were males and females aged between 13 and 15 years. Head teachers of 132 state secondary schools (i.e. schools with students in our age category) in 6 local education authorities in different parts of Scotland were contacted by email about the study. Schools were followed up by telephone calls if the head teacher (also called head master, Principal etc. in other countries) expressed interest in participating in the study. Recruitment stopped once six schools consented to participate.

For each participating school, study information booklets were distributed to all parents/carers of all eligible students on the school roll, including a form that could be returned if the parent/carer did not assent to their child’s participation in the study. Only students who are opted out of the study by a parent/carer were excluded. Students who have insufficient English language were not excluded because the Education (Additional Support for Learning) (Scotland) Act 2004 entitles those students to additional support for learning. Contact details for the research team were included in the booklet, allowing parents/carers the opportunity to contact the research team if they wished to discuss the study. Students whose parents did not opt them out of the study were provided with a verbal overview of the study by a researcher, a study information booklet and consent form. For those students who were opted out of the study by parents/carers and those that did not consent to participate, alternative educational opportunities were provided by the school whilst their classmates participated in the study.

### Intervention description

The intervention being tested was a refinement of our previous intervention [28]; two intervention components were the same (Components 1 and 2) and our previous study shows that these components improve sun safe intentions [28]; the third additional component was novel and had not been previously tested. The intervention was designed in accordance with two theoretical models - CSM and HAPA. The intervention was developed to address social cognitions by changing beliefs about skin cancer, evoke an emotional response to skin cancer, and shift sun protection intentions to actual behaviour by including action and coping planning.

*Component 1:* Information delivered during a presentation was designed to address key CSM dimensions (cause, consequence, identity, risk perception, controllability) and included information about personal experiences of skin cancer, incidence patterns, risk factors (e.g. when during the day risk of overexposure is greatest in the UK), associations between disease staging and survival, and benefits of SSE. The presentation also briefly touched on appearance e.g., concepts of beauty in relation to tanned and pale skin and the importance of some sun exposure for vitamin D. A skin cancer nurse specialist delivered the 50-min presentation on one occasion during the school day in a classroom or hall. After playing a 5-min film ‘Dear 16-year-old me’ (http://dcmf.ca), the nurse delivered the presentation with the aid of Microsoft PowerPoint slides. A Manual of the presentation that was developed by the research team was used as a guide for delivering the same presentation each time. A young adult skin cancer survivor (in this study the individual was male and hence, male pronoun is used) gave a brief 5-min talk after the nurse-delivered presentation. The talk was about his personal experience of melanoma diagnosis at 16 years old, impacts on his life and his views on sunscreen use and SSE. The film and the young person’s talk aimed to evoke an emotional response to skin cancer.

*Component 2:* This intervention component was based on the HAPA and aimed to shift intentions to use sun protection and conduct SSE by making plans to conduct these behaviours. A booklet with instructions to write sun protection and SSE action plans was handed to students at the end of the presentation. The booklet also included information about sunscreen use and SSE. Adolescents were asked to complete an action plan for regular monthly sunscreen use and an action plan for SSE in their own time as home. The SSE component of the booklet for instance, had three sections: a) information on the importance of planning; b) instructions of what should be included in the plan; c) formulating ‘if-then’ action plans (e.g., If I am having a shower then I will check my skin) and coping plans (e.g. To make sure I don’t forget, I will add the appointment to my calendar and put a reminder post-it on the fridge).

*Component 3:* Automated text messages were delivered on two days of the week for seven weeks after the 50-min presentation. All messages were sent during the summer holiday period relating to the schools in the study. This is because the summer holidays coincide with UK warm weather when there is the greatest risk of overexposure to the harmful effects of the sun. It is also when students may travel abroad to countries with hot climates. Messages were developed by the study investigators to apply to key theoretical CSM dimensions and address appearance. A total of 14 messages were developed. These messages were tailored to the target audience following feedback from a focus group of students (*n* = 13) who attended one of the participating schools. Participants were shown the messages that the study investigators developed and were asked to provide feedback, including how to make the text messages more likely to motivate themselves to protect their skin. Messages that the participants indicated that they did not like were removed from the list or were revised based on specific suggestions (Additional file 1). Messages contained information around sun safety behaviours and information about the effects of excessive exposure to the sun (Table 1). Messages were scheduled in the morning on a Monday and Friday.

**Table 1:** Text messages and relevant CSM dimensions

#	Text	CSM dimensions
Intro	Schools out for summer! This is Sunny Day, you will receive texts from me over the summer holiday as part of the sun safe study! We hope the information will help you stay safe in the sun!	
1	Fake it! If you like looking tanned then fake tan is better for your skin than sunbathing.	Risk perception
2	Spot the odd one out. To stay sunsafe: use sunscreen, wear sunglasses, wear a hat, cover up, stay in the shade, start a stamp collection.	Controllability
3	Skin cancer is the 2nd most common cancer in young adults in the UK. Reduce your chances of skin cancer by staying safe in the sun.	Risk perception Controllability
4	The sun can cause skin cancer through invisible rays called UV rays. You can’t see them but it is these rays that hit our body and cause skin cancer.	Cause
5	The two types of UV rays that affect our skin are UVA and UVB rays. UVA causes aging. This means that you will get wrinkles younger and your skin can turn leathery. Easily remembered as UVAgeing.	Consequence Appearance
6	UVB causes your skin to burn. Easily remembered as UVBurning.	Consequence
7	Ralf was diagnosed with skin cancer when he was 24. He said: “I should have protected my skin from the sun a lot more when I was young.”	Risk perception Controllability
8	Those who get diagnosed with skin cancer often regret not having been more careful when they were young. You need to be sunsafe now.	Consequence Timeline
9	“Sunscreen is smelly and sticky” – is that a good enough reason to risk getting sunburnt and increasing your chance of skin cancer?	Consequence Cause
10	You look so nice and healthy with your tan. Isn’t it weird that a sign of skin damage is seen as something healthy? A tan is skin damage.	Identity Appearance
11	Fake it! If you like looking tanned then fake tan is better for your skin than sunbathing.	Controllability Appearance
12	Avoid the lobster look. Sunburn hurts, makes you look stupid and keeps you out of action.	Consequence Appearance
13	You wouldn’t sit in a car without a seatbelt on, would you? Make sure you do the same in the sun. Sunscreen is your sun-seatbelt!	Controllability
14	How do you think celebrities keep their skin looking young? Thats right its sunscreen! Cheaper and less painful than a facelift…	Appearance Controllability
End	Thanks for taking part in our study – hope you had a good summer holiday!	

### Assessing intervention reach, adherence, impact, acceptability

#### Reach and adherence

Intervention reach was objectively measured using school attendance records. Reach was defined as the proportion of students on the school register who attended the presentation and were given the booklet (intervention components 1 and 2) and gave their mobile number (component 3) at the time they consented to the study. Adherence was self-reported by students at follow up and defined as the proportion of consenting students who received the presentation (component 1), read the booklet (component 2), and received text messages (component 3). Questions were: *‘Did you listen to the presentation about sun safety that [name of presenters] did?’ ‘Did you read the booklet that was handed out about sunscreen and skin self-examination?’ ‘Did you give your mobile phone number and receive text messages about sun safety over the summer holidays?’ Responses were yes, no and don’t know.*

#### Impact

Five-point continuous rating scales were used to assess students’ views about the impact of each intervention component. For example, to assess the perceived impact of component 3 (text messaging) 2 items were used to assess perceived impact: i) *‘On a scale of 1 to 5, did the text messages about sun safety increase the ways that you protected your skin from the harmful effects of the sun? e.g. using sunscreen, staying in the shade etc.’* ii) *‘On a scale of 1 to 5, did the text messages about sun safety influence whether you examined your skin for signs of possible skin cancer?’* Students answered each question using a scale of 1 (definitely did) to 5 (definitely did not).

#### Acceptability

Focus groups to elicit adolescents’ views on the acceptability of the intervention were conducted approximately 12 weeks after Component 1 of the intervention in each of the four intervention group schools. Focus groups were audio-recorded and took place during school time, in a classroom, at a time and place selected by the teacher and lasted approximately 20 min. Confidentiality was explained and informed consent was obtained in writing.

### Variables and measures

Outcome variables were measured before the school summer holidays in June (baseline) and after the summer holidays in September (follow-up). This is because the summer holidays coincide with UK warm weather when there is the greatest risk of overexposure to the harmful effects of the sun. Student responses were paired between the two timepoints via use of unique identifier after all questionnaires had been anonymised. Objective measures of sunburn and tanning were collected by two researchers in the classroom. A self-completed pen and paper questionnaire was completed by students in the classroom. Items for the self-report questionnaire were recommended by an international working group to measure sunburn and sun protection behaviours [46] and/or used in our previous study [28].

#### Objective measures of skin colour

The colour of our skin is made up of many different components including melanin (protective tanning pigment) and redness (erythema). The Mexameter (EnviroDerm MX18) is a spectrometer measurement technique, based on light reflection and absorption which measures melanin and erythema in the skin. The probe emits three wavelengths of light, chosen to correspond to the different absorption rates of melanin and haemoglobin. The feasibility of objectively measuring sunburn and tanning was assessed by measuring skin colour using a Mexameter, giving a “melanin index” calculated from the intensity of the absorbed and the reflected light at 660 and 880 nm and an “erythema index” from 568 and 660 nm [47]. Data suggest that the Mexameter can reliably detect erythema from exposure to 1.5 times the minimal erythemal dose (MED) of UV radiation; MED is determined by skin colour with paler skin being more sensitive to UV [48].

Three readings were made, each taking only a few seconds, on the right or left dorsal forearms (likely to be exposed to UV radiation) and behind the left or right ear (unlikely to be exposed to UV radiation). An increase in scores indicates more sun exposure between baseline and follow up.

#### Self-reported sunburn, severity and body location

Self-reported sunburn was measured using one item: *‘For people with white skin, sunburn is red skin that appears a few hours after being out in the sun and then fades after a few days. For people with naturally dark skin, sunburn is less visible but the skin feels hot in the sun and stays hot and is painful afterwards for a few days. During the last summer holidays, how many times did you have a red OR painful sunburn that lasted a day or more?* Students had nine options to choose to report how many times they had sunburn from 0 to ≥8.

Sunburn severity was measured using one item: *‘Which one of the following best describes your worst case of sunburn during the last summer holidays?’* Students had seven options to choose how to report severity: ‘Skin got hot and stayed hot for a couple of days, Skin went pink or slightly red, Skin went red but not sore, Skin went red and sore, Skin went red, sore and blistered, or I did not get sunburnt during the summer holidays.’

Body location was measured using one item: *‘Where on the body was your worst case of sunburn during the last summer holidays?’* Students had seven options to choose to report where on the body their worst case of sunburn occurred: ‘back, chest, leg or foot, arm or hand, shoulder or neck, head or face, or I did not get sunburnt during the summer holidays.’

#### Self-reported tanning

Three items were used to measure tanning: i) *‘Last summer did you get a suntan?’* Students had three options: ‘yes, no or don’t know’; ii*) ‘How many days did you sunbathe last summer to try to get a suntan? (by sunbathe, we mean that you stayed out in the sun because you wanted your skin to go browner or more golden in colour).* Students had four options ‘0 days, 1 to 5 days, 6 to 10 days, 11 or more days’. iii) *‘At present, do you use a sun-tanning bed (either at home, in a spa or a tanning shop on high street)’*. Students had three options: ‘yes, no or don’t know.’

#### Sun protection behaviours

Four items were used to measure sun protection behaviours: ‘*For the following questions, think about what you did when you were outside during the last summer holidays on a warm sunny day: i)* How often did you wear SUNSCREEN? ii) How often did you wear a SHIRT WITH SLEEVES that cover your shoulders? iii) How often did you stay in the SHADE or UNDER AN UMBRELLA? iv) How often did you wear SUNGLASSES?’ Students had four options: ‘never, rarely, sometimes, often’.

#### Skin self-examination (SSE)

One item was used to measure SSE: *‘In the past month, have you examined your skin for signs of possible skin cancer?’* Students had three options: ‘yes, no or don’t know.’

#### Social-demographic characteristics

Socio-demographic questions were included to gather data on age, gender and ethnicity.

### Analyses

As this was a feasibility study, the quantitative data were analysed using descriptive statistics. Baseline measurements were reported as n (%) for categorical data and mean (standard deviation) for continuous variables. The melanin and erythema indices were summarized as the arithmetic mean of the three readings taken on the forearm, and the arithmetic mean of the three readings taken behind the ear. Changes in the outcome measures were analysed within individuals (paired analysis) and reported in cross-tabulations (pre- and post- intervention) for categorical variables and as mean (standard deviation) of within-individual changes for continuous variables.

The control group was included in the study solely to determine the feasibility of recruitment of such a group. Thus, the more detailed results reported in this manuscript only include participants in the intervention group, to describe the outcomes pre- and post-intervention and the participants’ subjective views of the intervention impact. Only complete data (i.e., individually paired baseline and follow up sampled data) are included.

Audio-recorded qualitative data from focus groups were transcribed verbatim and analysed thematically using the Framework approach [49]. Qualitative findings provided contextual and explanatory understandings of adolescents’ experiences of the intervention.

## Results

### Recruitment, participant characteristics and data availability

Initially eight out of 130 schools in Scotland indicated an interest in participating in the study. Three schools had difficulty in facilitating the study within the timetable. There were four schools allocated to the intervention group and one school allocated to the control group. As previously pointed out, randomisation as a method for reducing bias was not used because we were not measuring effect in this feasibility study.

The school registers across all sites (intervention and control) indicate 724 students were eligible. No students declined to participate but some were either absent on the days the research was conducted or in a class that was not included in the study (a teacher could decide if their class was to be included in the study without giving any reason) and therefore 487 students consented to the study at baseline (67%), of which 385 (79%) were in the intervention group and 102 (21%) in the control group. Four parents/carers opted their child out of the study.

The characteristics of the study group are shown in Table 2. On average the intervention group were statistically significantly older than the control group (average age = 14.3 compared to 13.4) and had slightly more male participants (although this difference was not statistically significant). The distribution of ethnic groups was similar in the intervention and control groups, with the majority of the participants from the White ethnic group.

**Table 2:** Study group characteristics

Variable		Intervention		Controls	
		No.	*%*	No.	*%*
Age at start:	13	0	*(0%)*	57	*(58%)*
	14	234	*(66%)*	40	*(41%)*
	15	118	*(34%)*	1	*(1%)*
	Missing	33		4	
Gender:	Male	179	*(49%)*	42	*(44%)*
	Female	185	*(51%)*	53	*(56%)*
	Missing	21		7	
Ethnic group	White	358	*(93%)*	95	*(93%)*
	Mixed	11	*(3%)*	3	*(3%)*
	Asian	6	*(2%)*	1	*(1%)*
	Black	5	*(1%)*	0	*(0%)*
	Other	4	*(1%)*	0	*(0%)*
	Missing	1		3	

Objective skin measurements were available for 255 (66%) of the intervention group at baseline and 237 (61%) of the group at follow up. In one intervention school, the researchers ran out of time and could not collect measures from all students. Complete self-report data in schools were available for 247 (64%) adolescents in the intervention schools (i.e. we could pair baseline and follow-up for analysis of change in self-report outcome measures). In the control school, objective skin measurements were available for 101 (99%) of the control group at baseline and 79 (77%) at follow up. Complete self-report data in the control group was 57 (56%).

**Fig. 1:**
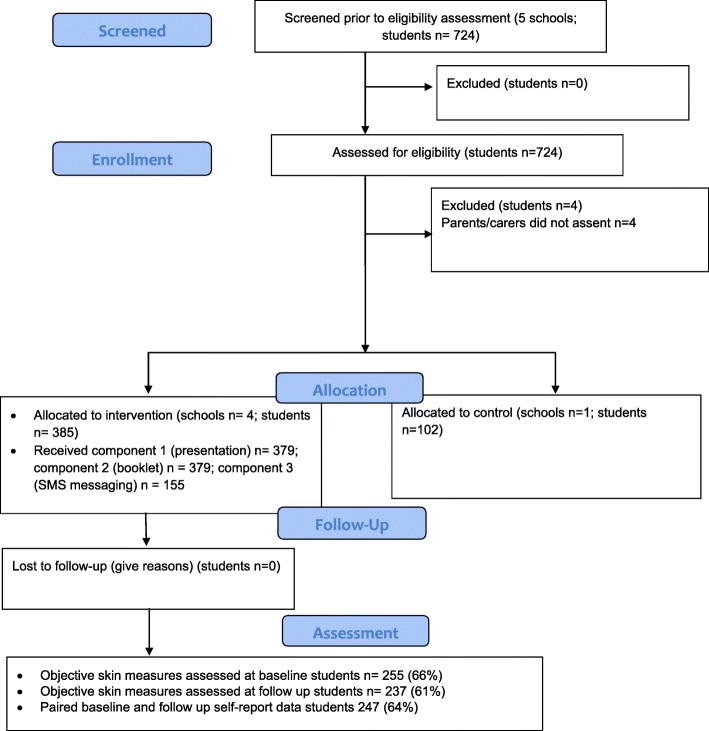
Study Flowchart

The study flowchart (Fig. 1) shows the number of students screened and assessed for eligibility, excluded, allocated to intervention or control group and the number of students in the intervention schools receiving the intervention and assessed at follow up. Given this is a feasibility study that is not designed to measure effect, only complete data for intervention schools is included in the results below.

### Intervention reach, adherence, relevance and acceptability

#### Reach and adherence

The number of intervention group students on the school register who attended the presentation about sun protection (component 1) and were given the booklet (component 2) was: 52%, *n* = 110; 72%, *n* = 93; 91%, *n* = 29; 84%, *n* = 147 for each intervention group school, respectively. The number of students on the school register who gave their mobile number to the research team (component 3) when consenting to the study was: 24%, *n* = 51; 16%, *n* = 21; 31%, *n* = 10; 41%, *n* = 73 for each intervention group school respectively.

Using self-report data, in total, 261 (92%) intervention group students listened to the presentation, 186 (68%) read the booklet with action plans, and 109 (39%) received text messages. Just over one quarter of the group (27%) received all three intervention components. Focus groups with some participants provide some indication for low adherence; for example, one student had changed his mobile phone number and so did not receive text messages and some students were concerned about their number being shared and receiving spam messages.

#### Impact

Table 3 summarises intervention group students’ perceived impact of each intervention component for sun protection and SSE. Only students who reported receiving the component and completed the question about perceived impact are reported. The most popular response in each category was the middle one (3), recorded by between 30 and 43% of participants. In general, students perceived the intervention impacted their sun protection behaviours more than SSE with no intervention component perceived as more impactful than another.

**Table 3:** Subjective response to intervention components

	Definitely did								Definitely did not	
	1		2		3		4		5	
	n	*%*	n	*%*	n	*%*	n	*%*	n	*%*
Component 1 (Presentation):										
Increase sun protection	36	*(14%)*	63	*(24%)*	88	*(34%)*	50	*(19%)*	21	*(8%)*
Skin examination	24	*(9%)*	52	*(20%)*	83	*(32%)*	40	*(15%)*	61	*(23%)*
Component 2 (Booklet):										
Increase sun protection	15	*(8%)*	46	*(26%)*	57	*(32%)*	45	*(25%)*	16	*(9%)*
Skin examination	14	*(8%)*	36	*(20%)*	53	*(30%)*	44	*(25%)*	31	*(17%)*
Component 3 (Text messages)										
Increase sun protection	17	*(16%)*	15	*(14%)*	46	*(43%)*	15	*(14%)*	14	*(13%)*
Skin examination	13	*(12%)*	13	*(12%)*	41	*(38%)*	24	*(22%)*	16	*(15%)*

#### Acceptability

Focus groups after the intervention were conducted in 3 intervention schools with 42 students participating across the sites. The main theme was *relevance*. Adolescents did not perceive melanoma risk as a major issue for their age group and because of UK weather. A summary of key points is presented for each of the three intervention components:

Component 1 (presentation): Most students said during the focus group that listening to the young adult skin cancer survivor talk about his experience of being diagnosed and treated for skin cancer was the best feature of the presentation. Students could recall what he said. They said that they could relate to him because he was a young person. They believed that the message about sun protection was more powerful coming from someone their age. Students questioned the relevance of a sun protection intervention during adolescence because they associated skin cancer with older people and did not perceive sun protection a priority at this stage in their lives. Further, they did not perceive that they were at skin cancer risk because of the UK weather. Students were able to recall key sun protection information delivered during the presentation including using sunscreen and wearing clothing to protect themselves from the harmful effects of the sun.

Component 2 (action planning): Students said that they did not complete the action plans for sunscreen use or SSE. Some students were unclear about the purpose of doing such detailed action plans for one behaviour. Nonetheless, some students liked receiving the booklet because it had information about sun protection and SSE that they could refer to at a later date should they wish to do so.

Component 3 (text messages): Students said that text messages acted as a regular reminder to use sun protection. Students preferred texts with specific advice about how to be safe in the sun and ways of obtaining a tan without sunbathing as opposed to just information about the dangers of UV radiation. Text messages that would make them laugh were welcomed. Students recommended visual examples to accompany text and recommended sending texts when relevant e.g., on sunny days. Some students questioned the relevance of sun protection messages in the UK because of the weather and perceived them as only relevant when they were in countries with regular sunshine.

### Melanin, erythema, sunburn, tanning, sun protection behaviours, skin self-examination

#### Melanin and erythema

The change in the melanin and erythema indices across the intervention period are summarised in Table 4 for the intervention group. A positive difference indicates an increase in the index. Indices of melanin increased on both the arm and the ear, with higher increases on the ear. Indices of erythema decreased on both sites.

**Table 4:** Change in melanin and erythema indices for intervention group

Site	Change in index over the intervention period	
	Mean	Standard deviation
Melanin (Arm)	17.0	*(63.2)*
Melanin (Ear)	45.1	*(74.2)*
Erythema (Arm)	−11.9	*(60.8)*
Erythema (Ear)	−20.0	*(62.8)*

#### Sunburn

Table 5 shows the numbers of self-reported occurrences of sunburn before and after the intervention. The numbers before the intervention refer to the number of occurrences in the previous summer and the numbers after the intervention refer to the number of occurrences during the intervention period. Due to small numbers, those reporting 4, 5, 6, 7 or 8 or more occurrences have been grouped together as 4 or more occurrences.

**Table 5:** Self-reported occurrence of sunburn comparing pre- and post-intervention scores

Pre-intervention	Post-intervention					
	No sunburn	1	2	3	4 or more	Total
No sunburn	38	17	4	1	0	60
1	25	20	16	5	0	66
2	8	15	21	11	3	58
3	2	8	12	3	8	33
4 or more	1	4	9	7	9	30
Total	74	64	62	27	20	247

Table 5 shows for instance, that out of the 60 students who reported ‘No sunburn’ the previous summer holidays (pre-intervention), 38 reported ‘no sunburn’ during the current summer holidays (post-intervention), 17 reported 1 incidence of sunburn, 4 reported 2 incidences, 1 reported 3 incidences and 0 reported 4 or more incidences of sunburn. Overall, 37% of participants reported the same number of sunburns pre- and post-intervention, 26% reported an increased number of sunburns and 37% reported a decreased number of sunburns.

The extent of the self-reported worst case of sunburn is shown in Table 6. Only those who reported occurrence of sunburn in the previous question are included. The percentage of participants reporting more severe sunburn is lower post-intervention than pre-intervention; for example, 55% of students reported that skin went red and sore or skin went red, sore and blistered in the previous summer holidays (pre-intervention) compared to 43% during the current summer holidays (post-intervention).

**Table 6:** Extent of worst case of sunburn comparing pre- and post-intervention scores

Extent of sunburn	Pre-intervention		Post-intervention	
	n	*%*	n	*%*
Skin stayed hot for a couple of days	19	*(10%)*	22	*(13%)*
Skin went pink or slightly red	26	*(14%)*	31	*(18%)*
Skin went red but not sore	37	*(20%)*	42	*(25%)*
Skin went red and sore	74	*(41%)*	54	*(32%)*
Skin went red, sore and blistered	25	*(14%)*	19	*(11%)*
Total	181		168	

Typically, sunburn was experienced on the shoulder and neck (around 52% of participants) followed by the back (18%) both pre- and post-intervention.

#### Tanning

Overall, 62% of intervention group participants reported getting a suntan in the summer prior to the intervention and 66% reported a suntan in the summer of the intervention. Table 7 shows the self-reported numbers of days of sunbathing each summer to try to get a suntan.

**Table 7:** Self-reported number of days sunbathing pre- and post-intervention

Pre-intervention	Post-intervention				
	None	1–5 days	6–10 days	11+ days	Total
None	70	16	8	6	100
1 to 5 days	27	32	12	8	79
6 to 10 days	4	13	7	8	32
11 or more days	3	2	12	19	36
Total	104	63	39	41	247

Just over 50% of participants reported similar sun-tanning behaviour pre- and post-intervention, 23% increased the number of days of sunbathing and 25% decreased the number of days sunbathing.

Very few participants reported using a sun-tanning bed (Acts in 2008 in Scotland and 2010 in England and Wales were introduced to prevent people under the age of 18 from using sunbeds on commercial premises, by making it an offence for sunbed businesses to allow people access under the age of 18 to sunbeds on their premises) - 5 participants reported doing so pre-intervention, and 4 participants reported doing so post-intervention. None of the participants reported using a sun-tanning bed both pre- and post-intervention.

#### Sun protection

Table 8 shows the self-reported use of sun protection measures pre- and post-intervention. The table shows the number and percentage of participants who reported taking each sun protection measure either sometimes or often. There was little change in behaviours across the intervention period.

**Table 8:** Use of sun protection measures pre- and post- intervention

Sun protection	Pre-intervention		Post-intervention	
	n	*%*	n	*%*
Wore sunscreen sometimes/often	186	*(75%)*	170	*(69%)*
Shirt with sleeves sometime/often	144	*(58%)*	140	*(57%)*
Stayed in shade sometimes/often	135	*(55%)*	127	*(51%)*
Wore sunglasses sometimes/often	151	*(61%)*	141	*(57%)*

#### Skin self-examination

Only 7 (3%) of participants reported examining their skin for signs of possible skin cancer on a regular basis pre-intervention, rising to 26 (11%) of participants post-intervention.

## Discussion

We successfully recruited and retained five schools and allocated one school to a wait-list control group. Hence, this small feasibility study suggests that the use of a wait-list controlled study design is acceptable. In this feasibility study, head teachers were approached only once about the study by email; nevertheless, the poor initial response rate highlights that future research conducted in UK secondary schools may face recruitment challenges. The challenges of recruitment and data collection in schools are recognised internationally [50, 51] and therefore we believe that the low response rate in our feasibility study is likely related to the general practicalities of accommodating research in schools irrespective of country. Some head teachers indicated that they were not in a position to participate because there was no space left on the school timetable to accommodate the research. This feasibility study therefore suggests that research teams may have more success in recruiting schools if head teachers are approached prior to the finalisation of timetables, which are often set a year in advance. Other studies recommend approaching a relevant teacher (e.g. a teacher responsible for personal, social and health education) rather than the head teacher [51] and avoiding examination periods when students are either in exams or studying for exams [28].

The feasibility study shows that objective measurement of melanin and erythema by Mexameter before and after the school summer holiday is acceptable to adolescents and is feasible to collect in schools. Nonetheless, the procedure, while taking only a minute per student, does require planning so that all students in the study can have measures taken within the available time set for the research on the school timetable. The school summer holiday in the UK is when sun exposure is most likely to produce a change in melanin. The direction of change in indices of melanin was as expected, with mean melanin scores increasing over the school summer holidays. Indices of erythema scores decreased. Erythema is the initial inflammatory response in the skin and represents redness of haemoglobin [47]. Hence, erythema (visible redness) is more transitory lasting no more than three days. The study suggests that measuring change in erythema before and after a six week summer holiday may not yield relevant or meaningful data. Instead, future studies should consider measuring erythema immediately before and no more than two days after adolescents have been exposed to the sun and ideally measured on a sunny day when sun protection is recommended. This presents particular challenges in countries such as the UK because sunny days are not guaranteed.

Another purpose of the feasibility study was to evaluate intervention reach and adherence. Reach and adherence can impact on statistical power and interpretation of trial results, including under-estimating any efficacy. There is no consensus on the acceptable minimum adherence level in trials and no standardised approach to adherence measurement in the field of complex interventions [52]. A review of treatment adherence in public health research reported that only 27% of research checked adherence to protocol [53] and few prior sun protection intervention studies have assessed reach and adherence. In this feasibility study, intervention reach (i.e., the proportion of students on the school register who attended the presentation) varied considerably between schools. Low reach was primarily due to school-level factors, such as not releasing all classes of eligible students to attend the presentation. Self-reported intervention adherence varied for each of the three intervention components. Adherence to the presentation (92%) and the booklet (68%) is higher in comparison to a study that reported that less than half of online skin cancer risk-reduction modules were completed by young people (18–25 years) [54]. The study found that the intervention was more effective in young people who completed more of the modules, thus highlighting the importance of adherence [55]. In our study, 39 % of consenting students received SMS (component 3) and only 27% of consenting students received all three intervention components. Given the high level of ownership of a mobile phone in this age group [37] we expected adherence for this component of the intervention to be higher.

The feasibility study provides some insight for participant-level influences on poor adherence. Some students during focus groups for instance, perceived that sun protection text messages were irrelevant for those living in the UK because of the inclement weather and this may account for why some students did not proffer their mobile number. Indeed, a key finding from the focus groups is that adolescents did not perceive that a sun protection intervention was relevant for their age group or people living in the UK because of their perception of the weather. Lack of perceived relevance may explain why the majority of adolescences in the study were neutral in their response to whether the intervention influenced their sun protection behaviours and SSE. Adolescents tend to be less concerned with the distant future [56] when skin cancer is more likely to occur and do not perceive themselves to be at risk of getting skin cancer [39]. A recent qualitative study concluded that lack of knowledge about the long-term risks of sun exposure most likely contributed to the perception that susceptibility to, and severity of the risks of sun exposure is low in adolescents [57]. Hence, lack of perceived relevance is a key challenge for sun protection interventions, particularly in countries with similar weather to the UK.

One of the ways of assessing the feasibility of outcome measures is to describe the direction of change in outcomes from baseline to follow up. As already discussed above, indices of melanin increased in the direction as expected but erythema did not. Severity of the worst case of sunburn decreased. The study highlighted that sunburn occurred primarily on the shoulder and neck followed by the back. Previous research carried out in the UK suggests that the commonest sites for melanomas in England for females were on the lower limb (45% of melanomas in females) and males on the trunk (38% of melanomas in males) in keeping with Scottish data. Data about body sites for sunburn may contribute towards understanding the relationship between chronic and intermittent sun exposure and melanoma [58, 59]. Sun protection behaviour did not change and results on tanning behaviour were mixed. These mixed results are typical of the few sun protection education intervention studies that have been conducted with adolescents [31, 60–65]. For example, a study that used self-report to measure sun protection behaviours and intentions pre- and post- the delivery of 36 SMS over 12 weeks found significant changes in some behaviours/intentions e.g., use of sunscreen but not others e.g., seeking shade and intentional tanning [39]. A randomized controlled trial of an education intervention comprising three class-based sessions found that self-reported weekend sun protection behaviours improved but not weekday sun protection behaviours [66].

Studies that have examined mediator variables point to possible reasons why studies report mixed results and suggest that key risk behaviour theoretical constructs, such as setting goals and action planning for sun protection mediate intervention effects [28, 55]. Nonetheless, it is not possible from the current body of evidence of education interventions targeting adolescents to draw any firm conclusions about the effectiveness of sun protection interventions targeting adolescents. This is in part due to methodological limitations; hence, the conclusion drawn over a decade ago in 2004 from a systematic review of evidence of interventions to prevent skin cancer by reducing exposure to UVR remains fast; that is, evidence is insufficient to determine the effectiveness of interventions in secondary schools to reduce sunburn occurrence and severity and change sun protection behaviour [60].

### Strengths and limitations

This feasibility study provides new evidence regarding sun protection education interventions that was previously lacking internationally. In particular, it shows that objective measurement of melanin is feasible in schools and acceptable to adolescents. However, several limitations of the study must be noted. First, by definition this feasibility study was not powered to measure intervention effects on sunburn and sun protection behaviours. Second, the recruitment strategy was not designed to yield a representative sample. As a result, the sample consisted of a small number of mainly white adolescents and so may not generalize to populations with darker skin pigmentations. Third, the study was conducted in a country with inclement weather. Whether the findings would be similar in countries with different weather conditions in particular, those with hot and sunny climates, is unclear. Moreover, some UK summers are hotter have more cloud cover than others, which again may be a confounding factor. Fifth, future studies should plan to assess if a key intervention component has most effect. For example, this feasibility study suggests that students found the brief talk given by a young cancer survivor (component 1) more engaging than action plans for sun safety and SSE (component 2). In this feasibility study we used single item measures to assess adherence to each of the three intervention components (e.g. *‘Did you read the booklet that was handed out about sunscreen and skin self-examination?’*) and participants could respond with either ‘yes’, ‘no’ or ‘don’t know’. Future studies should consider requesting more detail about interaction with key intervention components in order to assess for example, if some parts of the booklet were read. Finally, given that one of the main findings of this feasibility study was adolescents’ perception that sun protection was irrelevant for their age group, the extent to which education interventions alone can reduce sunburn occurrence and sun protection behaviours is questionable.

## Conclusions

It is feasible to conduct a wait-list controlled trial of a sun protection education intervention and objectively measure melanin and measure self-reported sun protection behaviours and SSE in adolescents before and after the school summer holiday. A major challenge for sun protection intervention studies is that adolescents do not see the relevance of sun protection and SSE for their age group. Lack of perceived relevance may be compounded in countries with inclement weather.

## Supplementary information

**Additional file 1.** Text messaging.

## Data Availability

The datasets used and/or analysed during the current study are available from the corresponding author on reasonable request.

## Abbreviations

CMS: Common-Sense Model of illness representation and self-regulation
HAPA: Health Action Process Approach
SMS: Short messaging service
SSE: Skin self-examination
UVR: Ultra violet radiation

## References

[1] McKenzie RL, Liley JB, Bjorn LO (2009). UV radiation: balancing risks and benefits. Photochem Photobiol.

[2] Brown KF, Rumgay H, Dunlop C, Ryan M, Quartly F, Cox A, Deas A, Elliss-Brookes L, Gavin A, Hounsome L (2018). The fraction of cancer attributable to modifiable risk factors in England, Wales, Scotland, Northern Ireland, and the United Kingdom in 2015. Br J Cancer.

[3] Di Nuzzo S, Pavanello P, De Panfilis G (2009). Density and proportions of the epidermal T cell population in human sun-exposed skin differ from those in sun-protected skin: preliminary immunohistochemical study. Arch Dermatol Res.

[4] Vallejo-Torres L, Morris S, Kinge JM, Poirier V, Verne J (2014). Measuring current and future cost of skin cancer in England. J Public Health (Oxf).

[5] Gandini S, Sera F, Cattaruzza MS, Pasquini P, Picconi O, Boyle P, Melchi CF (2005). Meta-analysis of risk factors for cutaneous melanoma: II. Sun exposure. Eur J Cancer.

[6] Dennis LK, Vanbeek MJ, Beane Freeman LE, Smith BJ, Dawson DV, Coughlin JA (2008). Sunburns and risk of cutaneous melanoma: does age matter? A comprehensive meta-analysis. Ann Epidemiol.

[7] Olsen CM, Zens MS, Green AC, Stukel TA, Holman CD, Mack T, Elwood JM, Holly EA, Sacerdote C, Gallagher R (2011). Biologic markers of sun exposure and melanoma risk in women: pooled case-control analysis. Int J Cancer.

[8] Dixon H, Borland R, Hill D (1999). Sun protection and sunburn in primary school children: the influence of age, gender, and coloring. Prev Med.

[9] Saridi M, Toska A, Rekleiti M, Wozniak G, Liachopoulou A, Kalokairinou A, Souliotis K, Birbas K (2012). Sun-protection habits of primary students in a coastal area of Greece. J Skin Cancer.

[10] Reinau D, Meier CR, Gerber N, Surber C (2014). Evaluation of a sun safety education programme for primary school students in Switzerland. Eur J Cancer Prev.

[11] Wright CY, Albers PN, Oosthuizen MA, Phala N (2014). Self-reported sun-related knowledge, attitudes and behaviours among schoolchildren attending south African primary schools. Photodermatol Photoimmunol Photomed.

[12] Diffey BL, Norridge Z (2009). Reported sun exposure, attitudes to sun protection and perceptions of skin cancer risk: a survey of visitors to Cancer Research UK's SunSmart campaign website. Br J Dermatol.

[13] Coogan PF, Geller A, Adams M, Benjes LS, Koh HK (2001). Sun protection practices in preadolescents and adolescents: a school-based survey of almost 25,000 Connecticut schoolchildren. J Am Acad Dermatol.

[14] Schofield PE, Freeman JL, Dixon HG, Borland R, Hill DJ (2001). Trends in sun protection behaviour among Australian young adults. Aust N Z J Public Health.

[15] Kyle RG, Macmillan I, Forbat L, Neal RD, O'Carroll RE, Haw S, Hubbard G (2014). Scottish adolescents’ sun-related behaviours, tanning attitudes and associations with skin cancer awareness: a cross-sectional study. BMJ Open.

[16] Gould M, Farrar MD, Kift R, Berry JL, Mughal MZ, Bundy C, Vail A, Webb AR, Rhodes LE (2015). Sunlight exposure and photoprotection behaviour of white Caucasian adolescents in the UK. J Eur Acad Dermatol.

[17] Degenhardt L, Chiu WT, Sampson N, Kessler RC, Anthony JC, Angermeyer M, Bruffaerts R, de Girolamo G, Gureje O, Huang Y (2008). Toward a global view of alcohol, tobacco, cannabis, and cocaine use: findings from the WHO world mental health surveys. PLoS Med.

[18] Viner R, Chief medical officer (2012). Life stage: adolescence. Our Children Deserve Better: Prevention Pays.

[19] Kendler KS, Myers J, Damaj MI, Chen X (2013). Early smoking onset and risk for subsequent nicotine dependence: a monozygotic co-twin control study. Am J Psychiatry.

[20] Lostritto K, Ferrucci LM, Cartmel B, Leffell DJ, Molinaro AM, Bale AE, Mayne ST (2012). Lifetime history of indoor tanning in young people: a retrospective assessment of initiation, persistence, and correlates. BMC Public Health.

[21] Marks R (1987). Skin cancer--childhood protection affords lifetime protection. Med J Aust.

[22] Leventhal H, Phillips LA, Burns E (2016). The common-sense model of self-regulation (CSM): a dynamic framework for understanding illness self-management. J Behav Med.

[23] Schwarzer R (2008). Modeling health behavior change: how to predict and modify the adoption and maintenance of health behaviors. Appl Psychol.

[24] Hagger MS, Orbell S (2003). A meta-analytic review of the common-sense model of illness representations. Psychol Health.

[25] Miller SMD MA. C-SHIP: A cognitive-social health information processing approach to cancer. In: Krantz D, editor. Perspectives in Behavioral Medicine: Lawrence Erlbaum; 1998. p. 219–44.

[26] Lau RR, Hartman KA (1983). Common sense representations of common illnesses. Health Psychol.

[27] Sniehotta FF, Nagy G, Scholz U, Schwarzer R (2006). The role of action control in implementing intentions during the first weeks of behaviour change. Br J Soc Psychol.

[28] Hubbard G, Kyle RG, Neal RD, Marmara V, Wang Z, Dombrowski SU (2018). Promoting sunscreen use and skin self-examination to improve early detection and prevent skin cancer: quasi-experimental trial of an adolescent psycho-educational intervention. BMC Public Health.

[29] Cameron LD (2008). Illness risk representations and motivations to engage in protective behavior: the case of skin cancer risk. Psychol Health.

[30] de Vries H, Mesters I, van't Riet J, Willems K, Reubsaet A (2006). Motives of Belgian adolescents for using sunscreen: the role of action plans. Cancer Epidem Biomar.

[31] Schuz N, Eid M (2013). Beyond the usual suspects: target group- and behavior-specific factors add to a theory-based sun protection intervention for teenagers. J Behav Med.

[32] Lowe JB, Borland R, Stanton WR, Baade P, White V, Balanda KP (2000). Sun-safe behaviour among secondary school students in Australia. Health Educ Res.

[33] Wichstrom L (1994). Predictors of Norwegian adolescents' sunbathing and use of sunscreen. Health Psychol.

[34] Williams AL, Grogan S, Clark-Carter D, Buckley E (2013). Appearance-based interventions to reduce ultraviolet exposure and/or increase sun protection intentions and behaviours: a systematic review and meta-analyses. Br J Health Psychol.

[35] Langbecker D, Diaz A, Chan R, Marquart L, Hevey D, Hamilton J. Educational programmes for primary prevention of skin cancer. Cochrane Db Syst Rev. 2014;4.

[36] Dombrowski SU, O'Carroll RE, Williams B (2016). Form of delivery as a key ‘active ingredient’ in behaviour change interventions. Br J Health Psychol.

[37] ofcom. Children and Parents: Media Use and Attitudes Report. London; 2017.

[38] Finch L, Janda M, Loescher LJ, Hacker E (2016). Can skin cancer prevention be improved through mobile technology interventions? A systematic review. Prev Med.

[39] Hingle MD, Snyder AL, McKenzie NE, Thomson CA, Logan RA, Ellison EA, Koch SM, Harris RB (2014). Effects of a short messaging service-based skin cancer prevention campaign in adolescents. Am J Prev Med.

[40] van de Mortel T (2008). Faking it: social desirability response bias in self-report research. Aust J Adv Nurs.

[41] Geller AC, Dickerman BA, Taber JM, Dwyer LA, Hartman AM, Perna FM (2018). Skin cancer interventions across the cancer control continuum: a review of experimental evidence (1/1/2000-6/30/2015) and future research directions. Prev Med.

[42] Loscalzo J (2009). Pilot trials in clinical research: of what value are they?. Circulation.

[43] Thabane L, Ma J, Chu R, Cheng J, Ismaila A, Rios LP, Robson R, Thabane M, Giangregorio L, Goldsmith CH (2010). A tutorial on pilot studies: the what, why and how. BMC Med Res Methodol.

[44] Arain M, Campbell MJ, Cooper CL, Lancaster GA (2010). What is a pilot or feasibility study? A review of current practice and editorial policy. BMC Med Res Methodol.

[45] Gaglio B, Shoup JA, Glasgow RE (2013). The RE-AIM framework: a systematic review of use over time. Am J Public Health.

[46] Glanz K, Yaroch AL, Dancel M, Saraiya M, Crane LA, Buller DB, Manne S, O’Riordan DL, Heckman CJ, Hay J (2008). Measures of sun exposure and sun protection practices for behavioral and epidemiologic research. Arch Dermatol.

[47] Matias AR, Ferreira M, Costa P, Neto P (2015). Skin colour, skin redness and melanin biometric measurements: comparison study between Antera((R)) 3D, Mexameter((R)) and colorimeter((R)). Skin Res Technol.

[48] Qian CY, Yuan C, Tan YM, Liu XP, Dong YQ, Yang LJ, Wu PL, Wang XM (2015). Comparing performance of Chromameter(R), Mexameter(R) and full-field laser perfusion imaging for measurement of ultraviolet B light-induced erythema. Clin Exp Dermatol.

[49] Spencer L, Ritchie J, O’Connor W, Ritchie JL (2003). Analysis: practices, principles and processes. Qualitative Research Practice: A Guide for Social Science Students and Researchers.

[50] Bartlett R, Wright T, Olarinde T, Holmes T, Beamon ER, Wallace D (2017). Schools as sites for recruiting participants and implementing research. J Community Health Nurs.

[51] Testa AC, Coleman LM (2006). Accessing research participants in schools: a case study of a UK adolescent sexual health survey. Health Educ Res.

[52] Graham L, Wright J, Walwyn R, Russell AM, Bryant L, Farrin A, House A (2016). Measurement of adherence in a randomised controlled trial of a complex intervention: supported self-management for adults with learning disability and type 2 diabetes. BMC Med Res Methodol.

[53] Borrelli B (2011). The assessment, monitoring, and enhancement of treatment fidelity in public health clinical trials. J Public Health Dent.

[54] Heckman CJ, Handorf EA, Darlow SD, Ritterband LM, Manne SL (2017). An online skin cancer risk-reduction intervention for young adults: mechanisms of effects. Health Psychol.

[55] Darlow S, Heckman C (2017). Results from a tailored SMS and behavior-tracking pilot study on sun-safe behaviors in young women. Health Educ Behav.

[56] Loewenstein GF, Weber EU, Hsee CK, Welch N (2001). Risk as feelings. Psychol Bull.

[57] Fitch-Martin AR, Menger LM, Loomis AD, Hartsough LES, Henry KL. “We Don’t Really Do Anything Unless it’s Really Bad”: Understanding Adolescent Sun Protective Knowledge, Attitudes and Behaviors in the U.S. J Prim Prev. 2018. 10.1007/s10935-018-0515-x 30008040

[58] Wallingford SC, Alston RD, Birch JM, Green AC (2011). Increases in invasive melanoma in England, 1979-2006, by anatomical site. Br J Dermatol.

[59] MacKie RM, Bray C, Vestey J, Doherty V, Evans A, Thomson D, Nicolson M, Scottish Melanoma G (2007). Melanoma incidence and mortality in Scotland 1979-2003. Br J Cancer.

[60] Saraiya M, Glanz K, Briss PA, Nichols P, White C, Das D, Smith SJ, Tannor B, Hutchinson AB, Wilson KM (2004). Interventions to prevent skin cancer by reducing exposure to ultraviolet radiation: a systematic review. Am J Prev Med.

[61] Buendia Eisman A, Arias Santiago S, Moreno-Gimenez JC, Cabrera-Leon A, Prieto L, Castillejo I, Conejo-Mir J (2013). An internet-based programme to promote adequate UV exposure behaviour in adolescents in Spain. J Eur Acad Dermatol Venereol.

[62] White KM, Hyde MK, O'Connor EL, Naumann L, Hawkes AL (2010). Testing a belief-based intervention encouraging sun-safety among adolescents in a high risk area. Prev Med.

[63] Miljkovic S, Baljozovic D, Krajnovic D, Tasic L, Sbutega-Milosevic G (2014). The impact of education on Adolescents’ sun behavior: experiences from Serbia. Srp Ark Celok Lek.

[64] Olson AL, Gaffney CA, Starr P, Dietrich AJ (2008). The impact of an appearance-based educational intervention on adolescent intention to use sunscreen. Health Educ Res.

[65] Buller DB, Reynolds KD, Yaroch A, Cutter GR, Hines JM, Geno CR, Maloy JA, Brown M, Woodall WG, Grandpre J (2006). Effects of the sunny days, healthy ways curriculum on students in grades 6 to 8. Am J Prev Med.

[66] Passerini J, Romiti A, D'Antonio C, Mastromarino V, Marchetti P, Volpe M, Conti E (2012). Tailored angiogenesis inhibition in cancer therapy: respecting the heart to improve the net outcome. Current Signal Transduction Therapy.

