# Supplementary material for: Sun protection education for adolescents: a feasibility study of a wait-list controlled trial of an intervention involving a presentation, action planning, and SMS messages and using objective measurement of sun exposure
Source: BMC Public Health. 2020 Jan 30;20:131. doi: 10.1186/s12889-020-8265-0 (PMC6990594; doi:10.1186/s12889-020-8265-0)
Supplement: Supplementary file 1 — Additional file 1. Text messaging. [file 12889_2020_8265_MOESM1_ESM.docx]

| **Proposed text messages** | **Comments**  **Please circle the response that best reflects how you feel about the text message – is it like to help you or unlikely to help you?**  **Please add any comments you might have in the box next to the message** |
| --- | --- |
| We are coming to the end of summer - don’t be fooled some days the UV Index is still high. Check the weather in advance and apply sunscreen if you need too! | Likely or Unlikely |
| Do you know your skin type? Light coloured skin burns more easily. Make sure to protect yourself – check our app for tips! | Likely or Unlikely |
| Sunburn puts you at risk of developing skin cancer. Protect yourself with sunscreen, cover with clothing & seek shade | Likely or Unlikely |
| Its not only you who suffers if you develop skin cancer – what about your family? Protect yourself from the sun. | Likely or Unlikely |
| Sunburn puts you at risk of developing skin cancer. Protect yourself with sunscreen, cover with clothing & seek shade | Likely or Unlikely |
| When its hot out there – stay hydrated and drink plenty of water! | Likely or Unlikely |
| There’s no healthy way to suntan. Tanning = skin damage. Protect your skin | Likely or Unlikely |
| Healthcare professionals recommend applying sunscreen daily over summer to protect the skin from sun damage | Likely or Unlikely |
| Burnt skin puts you at a higher risk of developing cancer. Cover up with clothing & sunscreen | Likely or Unlikely |
| Sunscreen is not just for holidays – its for life! | Likely or Unlikely |
| Every day in summer, apply sunscreen to all sun-exposed areas of the body | Likely or Unlikely |
| Are you taking steps to protect yourself from the sun? Good work! | Likely or Unlikely |
| Taking sun protective measures will help reduce your chances of developing skin cancer | Likely or Unlikely |
| Excessive sun exposure ages your skin - protect is with sunscreen | Likely or Unlikely |
| The UV Index is high – seek shade during midday hours, cover up & wear sunscreen | Likely or Unlikely |
| Healthcare professionals recommend applying sunscreen daily over summer to protect the skin from sun damage | Likely or Unlikely |
| Every day in summer, apply sunscreen to all sun-exposed areas of the body | Likely or Unlikely |
| Sun’s out, guns out? Think again, protect your skin with sunscreen & cover up | Likely or Unlikely |
| Be strong – keep healthy and stay safe in the sun | Likely or Unlikely |
| Be wise – examine your skin regularly | Likely or Unlikely |
| You are smart – check your moles | Likely or Unlikely |
| Prevent your skin from being described as: “dry, cracked, scaly, blistered, and scarred.” Apply sunscreen daily! | Likely or Unlikely |
| YOU can do it! Avoid getting sunburnt. Use sunscreen. | Likely or Unlikely |
| YOU can do. It! Check your moles | Likely or Unlikely |
| The sun’s UV rays are strongest between 11am and 3pm – stay in the shade during this time | Likely or Unlikely |
| You don’t have to get blisters or peeling | Likely or Unlikely |
| Reduce your risk of skin cancer NOW - don’t get burnt | Likely or Unlikely |
| Sunburn in childhood increases skin cancer risk in adulthood | Likely or Unlikely |
| Detecting a skin cancer early enough will save your life | Likely or Unlikely |
| Too much sun = ageing skin and wrinkles | Likely or Unlikely |
| Too much sun = blotchy skin | Likely or Unlikely |
| Too much sun = sore skin | Likely or Unlikely |
| Too much sun = skin cancer | Likely or Unlikely |
| Tanning beds average 13 on the UV index, which puts you at risk for dry skin, nausea, premature wrinkling, skin infections, and skin cancer. | Likely or Unlikely |
| No sunburns = no pain, no hurting | Likely or Unlikely |
| Too much sun increases your risk of skin cancer | Likely or Unlikely |
| Most skin cancers are cured if discovered early, so please check your skin for irregular spots. Consult a doctor if you see something | Likely or Unlikely |
| Use factor 30 or above to avoid getting sunburnt | Likely or Unlikely |
| Look for changes in moles | Likely or Unlikely |
| Stock up with Factor 30 sunscreen during the summer months | Likely or Unlikely |
| Pop that sunscreen and your sunglasses in your bag | Likely or Unlikely |
| Re-apply sunscreen every 2-3 hours | Likely or Unlikely |
| Quickly check your moles each month when in the shower | Likely or Unlikely |
| Pop that sunscreen in the shopping basket | Likely or Unlikely |
| Playing football today? Slap the sunscreen on | Likely or Unlikely |
| Having a shower? Check your skin | Likely or Unlikely |
| Keep that sunscreen in your bag just in case you forget | Likely or Unlikely |
